# Discussions on Human Enhancement Meet Science: A Quantitative Analysis

**DOI:** 10.1007/s11948-025-00531-6

**Published:** 2025-02-05

**Authors:** Tomasz Żuradzki, Piotr Bystranowski, Vilius Dranseika

**Affiliations:** 1 Jagiellonian University, Institute of Philosophy & Interdisciplinary Centre for Ethics, ul. Grodzka 52, Kraków, 31-044 Poland; 2 Jagiellonian University, Interdisciplinary Centre for Ethics, ul. Grodzka 52, Kraków, 31-044 Poland; 3 Max Planck Institute for Research on Collective Goods, Bonn, Germany

**Keywords:** Enhancement, Biosciences, Topic modeling, Citation analysis

## Abstract

The analysis of citation flow from a collection of scholarly articles might provide valuable insights into their thematic focus and the genealogy of their main concepts. In this study, we employ a topic model to delineate a subcorpus of 1,360 papers representative of bioethical discussions on enhancing human life. We subsequently conduct an analysis of almost 11,000 references cited in that subcorpus to examine quantitatively, from a bird’s-eye view, the degree of openness of this part of scholarship to the specialized knowledge produced in biosciences. Although almost half of the analyzed references point to journals classified as Natural Science and Engineering (NSE), we do not find strong evidence of the intellectual influence of recent discoveries in biosciences on discussions on human enhancement. We conclude that a large part of the discourse surrounding human enhancement is inflected with “science-fictional habits of mind.” Our findings point to the need for a more science-informed approach in discussions on enhancing human life.

## Introduction

Advancements in genetic manipulation and related technologies have enabled researchers to alter and replace DNA in organisms, as well as transfer genetic material between organisms. The developments in stem-cell research, e.g., somatic-cell nuclear transfer, which moves genetic material from a donor egg to a denucleated egg, enabled the cloning of mammals in 1996. Other recent advancements in this area include the generation of inducible pluripotent stem cells (iPSCs) in the 2000s or mitochondrial DNA (mtDNA) transfer in embryos in 2013. Although the direct manipulation of the genome of living organisms (usually plants for agriculture) was mastered by scientists in the 1970s and 1980s, the development of the CRISPR/Cas9 method in 2012 was considered a revolution owing to its efficiency and cost effectiveness, and the rank of this discovery was confirmed by the Nobel Prize awarded to its creators, Emmanuelle Charpentier and Jennifer Doudna, in 2020. In 2014, CRISPR/Cas9 germline modifications were first used in nonhuman primates; in 2015, they were used in nonviable human embryos (Liang et al., 2015); and in 2018, a notorious experiment conducted in China resulted in the birth of the first genetically modified babies. Therefore, the use of CRISPR/Cas9 could lead to permanent, heritable changes in the human genome. Simultaneously, other technological developments (e.g., pharmaceutical, cybernetic, nanotechnological) are – or may soon be – used to challenge human physical, cognitive, or emotional limitations (for a recent overview, see Jotterand & Ienca, 2024).

Since its early development, bioethics has been treated as a link between science and humanities (Potter, 1971) and seems to be a discipline particularly suitable for quick reactions to developments in genetic manipulation and related technologies, delineated in the previous paragraph. Bioethics emerged as an institutionalized discipline in the 1970s in response to rapid changes in medical technologies and the need to regulate biomedical research. The first U.S. governmental bioethics commission was established as a result of research scandals in the 1960s and 1970s, during which scientists were experimenting on people without the latter knowing this. Thus, bioethics as a discipline was set up to recommend constraints on the activity of scientists: As the author of a book on the human gene-editing debate noted, the very “origin of public bioethical debate itself was in the overreach of scientists and physicians, and the public calling for limits to avoid a dystopian future” (Evans, 2020). Accordingly, it is often claimed that the aim of bioethics as an academic discipline is not so much to broaden knowledge, but rather to present guidance (to researchers, policy-makers, and the broader public) regarding the current challenges in biotechnology and biomedical research. A recent paper on the translation of bioethics research states that the overarching goal of bioethics is “the translational process that advances academic endeavors aimed toward creating change in the world” (Mathews et al., 2016; see also Hofmann & Magelssen, 2018; Mertz et al., 2019). The book on the history and future of bioethics treats bioethicists as scholars who “engage in what political scientists would call ‘agenda-setting’ in the public sphere, defining the legitimate boundaries of public debates, identifying which ethical issues are ‘important’ and which are not” (Evans, 2012). The author highlights that bioethicists’ conclusions influence what actually happens in the real world through direct or indirect mechanisms (Evans, 2012).

Although radical human enhancement by genetic manipulation had been widely discussed for years before it became practically possible (Evans, 2002, 2020), one could expect recent scientific and technological developments in this area to have reignited debates in bioethics, especially in the context of prohibiting, limiting, and controlling some types of scientific research. The need for such discussions appears particularly pressing given the common expectations of rapid progress in human enhancement. For example, the authors of the executive summary of the U.S. National Science Foundation (NSF) comprehensive report detailing ethical issues arising from human enhancement emphasize that “With ongoing work to unravel the mysteries of our minds and bodies, coupled with the art and science of emerging technologies, we are near the beginning of the Human Enhancement Revolution” (Allhoff et al., 2011). Bostrom and Savulescu, two well-known enthusiasts of human enhancement, not only observe that “human enhancement has grown into a major topic of debate in applied ethics” but also claim that this interest “has been stimulated by advances in the biomedical sciences” (Bostrom & Savulescu, 2009). This tight connection with ‘good science’ is assumed to lie at the very heart of what is called ‘new’ or ‘liberal eugenics,’ in contrast with the ‘old’ and allegedly unscientific one (Sparrow, 2011).

The view expressed by Bostrom and Savulescu (2009) seems to be widely shared among bioethicists. More than a decade ago, John Harris claimed that “Moral enhancement is coming to the forefront of bioethical scholarship for an interesting combination of reasons” (Harris, 2011, p. 103). He observed that discussions on this topic combine “cutting-edge science with mainstream philosophy and with the hopes and fears of ordinary people” (Harris, 2011, p. 103; see also Bostrom & Savulescu, 2009; Sparrow, 2017; de Melo-Martín, 2022). In contrast, some other authors, often from outside of bioethics itself, complained that “the enhancement debate… suffers from a lack of empirical input” (Kourany, 2014) or that “the human enhancement discourse is now untethered from any specific technoscientific research programme” (Schick, 2016).

How can the scale, impact, and visibility of recent science and technology development on bioethical discussions on human enhancement be evaluated? Traditionally, such questions were addressed through ‘close reading’ of the relevant body of texts to determine the level of engagement of those texts with other disciplines. However, this approach is nontransparent, making it difficult to trace the evidence used to form definitive, albeit sometimes contradictory, conclusions, as seen in Harris (2011) and Kourany (2014). Moreover, this method often results in general claims based on arbitrary sampling from a large corpus (for more on the problems with ‘humanistic interpretation,’ see Pääkkönen & Ylikoski, 2021, p. 1465). In contrast to numerous analyses of ‘empirical bioethics’ itself, there are very few studies on how bioethicists use empirical data produced in biosciences to influence, for example, the regulations of scientific practice. In contrast, this paper addresses this question more systematically. First, we used a ‘distant reading’ approach based on topic modeling (a computational text-mining technique aimed at discovering hidden thematic compositions in large text corpora) to analyze the corpus of 19,488 bioethics papers published since 1971 in seven leading journals and addressing human enhancement.^1^ Second, with regard to the content of all the analyzed papers, we delineated a part of this corpus that addresses human enhancement. Third, we analyzed citation flows from that subcorpus to journals representing different scientific disciplines, in order to measure the degree of openness of this type of bioethics to the relevant specialized knowledge in biosciences.^2^

Thus, the motivation for this study comes from our expectation that conceptual, normative, or regulatory discussions in bioethics, which are strictly supposed to follow scientific discoveries, influence the regulatory framework of scientific research, indirectly also influencing the direction of developments in biosciences. To realize these aims, bioethics is most effective “when the practitioner is knowledgeable not only in the field of ethics, but also in the relevant scientific discipline, i.e., medicine, neuroscience, or some other field in the life sciences” (Tuana, 2010). We assume that this ‘knowledgeableness’ could be indirectly observed *via* the reference lists of bioethics publications, as bioethicists engaging with a given area of science and aiming at conducting a scientifically informed discussion can be expected to cite relevant scientific publications. If this were the case, one would observe a specific pattern of citations outgoing to the papers that are central to the enhancement debates in bioethics, for example frequent references to crucial contemporary studies from the biosciences (e.g., on CRISPR/Cas9).

In the next section, we show how our previous study that used topic modeling (Bystranowski et al., 2022) is useful for delineating and analyzing discussions on human enhancement in bioethics. In the following sections, we check how closely these discussions follow scientific breakthroughs in this regard and discuss our results.

## Bioethics and Human Enhancement

### Methodology: Topic Modeling

For the purposes of training our topic model (Bystranowski et al., 2022), we defined the broadly understood discipline of bioethics (including philosophy of medicine) as the content published by the journals that the researchers themselves find the most influential for this discipline. To identify the list of the most representative journals, we invited experts to provide a list of key journals, and the survey resulted in the selection of seven journals.^3^

We then collected a complete corpus of all the texts published in these journals (19,488 texts published from 1971 until early 2021), and we used an unsupervised machine-learning algorithm to identify 91 meaningful topics representing distinct themes of interest in the discipline in this corpus. The latent Dirichlet allocation (LDA) algorithm, which we used, identifies *topics*,^4^ that is, probability distributions, ascribing high probability to terms that tend to cluster together across documents in a given corpus (Blei et al., 2003). Those topics are characterized by relatively small sets of terms that tend to cooccur in the same documents, where each of the documents – as is assumed – is composed of a relatively small number of topics.^5^ Thus, it is typically easy to *interpret* topics, that is, to understand sets of terms as distinctive themes discussed in the analyzed corpus (for more on humanistic interpretations of unsupervised machine-learning methods, see Pääkkönen & Ylikoski, 2021). In our previous studies, we used topic modeling to analyze how various topics are related to one another or how they change over time (Bystranowski et al., 2022), and to analyze intellectual trends over the first five decades of the *Journal of Medical Ethics *(Dranseika et al., 2025). In this study, we use this method to delineate a subcorpus of papers that discuss problems related to human enhancement.

In addition to the high-probability terms associated with the topics (see Table 1), researchers’ interpretations may also focus on documents that are the most characteristic of the topics. For example, if the model’s output includes a topic characterized by the terms ‘enhancement’, ‘enhance’, ‘technology’, ‘intervention’, ‘cognitive’, ‘capacity’, ‘trait’, ‘morally’, ‘improve’, and ‘bioenhancement’, one can reasonably interpret such a *topic* as being connected to debates about human enhancement, particularly cognitive or moral enhancement (we labeled it *(Moral) Enhancement*). To corroborate this interpretation, one can also easily check that the documents most characteristic of this topic indeed concern a *moral* type of enhancement.^6^ The most characteristic paper for the topic *(Moral) Enhancement* is the paper ‘Engendering moral post-persons: A novel self‐help strategy’, published in the journal *Bioethics* in 2020. It argues that “we should bring about moral postpersons” as moral exemplars (Crutchfield, 2020). Six out of ten most characteristic papers for this topic discuss (or at least cite) the book *Unfit for the future. The need for moral enhancement* by Persson and Savulescu (2012), five of them have the phrase ‘moral enhancement’ or ‘moral bioenhancement’ in their titles, and two papers comment on the target article ‘Egalitarianism and moral bioenhancement’ by Robert Sparrow, published in *AJOB* in 2014, which discusses normative assumptions around “the society-wide program of biological manipulations required to achieve the purported goals of moral bioenhancement” (Sparrow, 2014).

To analyze the ways in which bioethics reacts to biomedical discoveries important for human enhancement, we decided to start our new analysis with the topic *(Moral) Enhancement*. Our previous study (Bystranowski et al., 2022) showed that this topics has enjoyed the greatest relative growth (mean prominence from 0.03% in 1976-80 to 0.97% in 2016-20) in the corpus, which confirms the commonly expressed intuition that discussions on enhancement are gaining popularity in bioethics.^7^ We then checked four additional topics denoting distinct areas of research present in the target journals with which this topic is most strongly correlated^8^: *Germline (Germline modification and gene therapy)*, *Ecology (Conservation and ecology)*, *Offspring (Obligations to offspring)*, and *Genetics (Genetics: concepts and research)*.^9^ To delineate a subcorpus of 1,360 documents that discuss problems related to scientific modification of life, particularly human life, we chose the papers that are the most characteristic for these five topics,^10^ which we treat as the ENHANCEMENT (ENH) cluster.^11^ Notably, the novelty of our method of delineating the subcorpus is based on the very content of the analyzed papers and not their reference list (as in bibliographic coupling, which connects articles that cite the same sources; see Kessler, 1963) or the list of citing articles (as in a co-citation network that links articles if they are cited by the same sources, see Small, 1973); for a discussion on other types of bibliometric delineations of scientific topics or fields, see Zitt et al., 2019).

### The ENHANCEMENT Cluster: What is it About

In this section, we briefly describe the resulting ENHANCEMENT cluster. We understand the topic of *Germline* as concerning mostly issues related to the ethics of human gene-editing. This interpretation was corroborated by the most characteristic paper for this topic, which is a target article, ‘Revising, correcting, and transferring genes’, published in *AJOB* in 2020, and the top 10 papers include two commentaries to this article (Cwik, 2020). Most papers from this set discuss the very distinction between germline and somatic gene-editing; four papers focus on the CRISPR/Cas9 method of gene-editing, and two focus on modifications of the mitochondrial genome. The topic most strongly correlated with *Germline* is *Offspring*, which mostly concerns the duties of prospective parents toward their possible future children. The most characteristic paper, ‘Do we need an alternative ‘relational approach’ to saviour siblings?’ (Wilkinson, 2015), is a part of the ‘Author meets critics’ discussion on the book *Saviour siblings: A relational approach to the welfare of the child in selective reproduction* (Taylor-Sands, 2013). Seven out of the 10 most characteristic papers discuss (or at least mention) the nonidentity problem (NIP); five papers discuss the well-known article ‘Procreative beneficence. Why we should select the best children’ published in the journal *Bioethics* by Savulescu (2002a). We interpreted the topic *Genetics* (which shares its first two characteristic terms, that is, ‘genetic’ and ‘gene’, with the topic *Germline*) as concerning mostly the complicated relationships between genetics and human behavior. The most characteristic document, ‘Why is studying the genetics of intelligence so controversial?’, was published in *HCR* (Tabery, 2015), and many other papers in the top 10 for this topic address behavioral genetics and genetic prediction of cognitive ability. Other papers in this set discuss the human-genome project or the meaning and historical development of the concept of ‘genes’. Finally, at first glance, the topic *Ecology* (closely correlated with *Genetics* and *Germline*), characterized by the terms ‘natural’/’nature’, ‘species’, ‘biological’/’biology’, ‘environment’/’environmental’, ‘organism’, and evolutionary/evolution, does not seem to fit well into this cluster. However, in this case, checking the most characteristic papers was particularly helpful in interpretation. The most characteristic paper, ‘De-extinction and conservation genetics in the anthropocene’ (together with 7 other papers from the top 10), was published in the *HCR* as part of a special issue on the de-extinction of some species of animals (Sandler, 2017). Additionally, one characteristic paper for this topic discusses the limits of ‘biotic ethics’, which concentrates on organic gene/protein life, in the context of human expansion in space that can transform our understanding of life (Mautner, 2009). Therefore, in this case, it seems that interest in genetics is crucial to understanding the link between this topic and the rest of the ENH cluster.

**Table 1:** The list of topics in the ENHANCEMENT cluster and their prominence in the corpus

Short label	Long label	Top-10 terms	Corpus prominence %	Corpus prominence in 2011-20 %
*Enhancement*	*(Moral) enhancement*	enhancement enhance technology intervention cognitive capacity trait morally improve bioenhancement	0.62	0.97
*Germline*	*Germline modification and gene therapy*	genetic gene therapy clone cloning disease intervention technology engineering germline	0.57	0.64
*Ecology*	*Conservation and ecology*	natural nature specie biological environment environmental organism evolutionary evolution biology	0.47	0.53
*Offspring*	*Obligations to offspring*	child parent genetic choice pgd reproductive future harm selection bear	0.78	0.81
*Genetics*	*Genetics: concepts and research*	genetic gene genomic genome dna disease trait sequence environmental factor	0.66	0.67

Our online supplementary materials enable additional analyses, e.g., to analyze the number of texts within the ENH corpus broken down by year, the volume of the five topics in the corpus in absolute terms, the number of articles from the ENH corpus in seven main bioethics journals, etc.^12^

## Do Enhancement Debates Follow Science?

### Methodology: Citation Analysis

In this section, we analyze the citations outgoing from documents in the ENH subcorpus. We assume that, by analyzing the citation flow, we can identify some core ideas and concepts in the ENH subcorpus and their genealogy. Using bibliometric methods, we answer the question of which parts of biosciences have privileged ties with bioethics. To what degree are bioethics debates about enhancement empirically informed? Which discoveries from biosciences had an impact on bioethics debates?

To achieve this aim, we combined the reference lists from 1,360 documents belonging to the ENH subcorpus and published between 1971 and 2021. We used the Web of Science (WoS) citation database. This resulted in the list of 10,946 citations from the ENH subcorpus to articles published in journals indexed by WoS (both the Core Collection and Emerging Sources Citation Index – ESCI). For the disciplinary classification of journals, we refer to the three-level U.S. National Science Foundation classification, the advantage of which (in contrast to WoS or Scopus categorizations) is that it assigns each journal to a single discipline.^13^ We modified the NSF classification by adding the discipline of BIOETHICS,^14^ defining it as publications in 21 specific journals indexed by WoS (7 journals covered in the topic-modeling study we refer to, plus an additional 14 included by our expert judgment).^15^

### What is Cited in the ENHANCEMENT Subcorpus

**Fig. 1:**
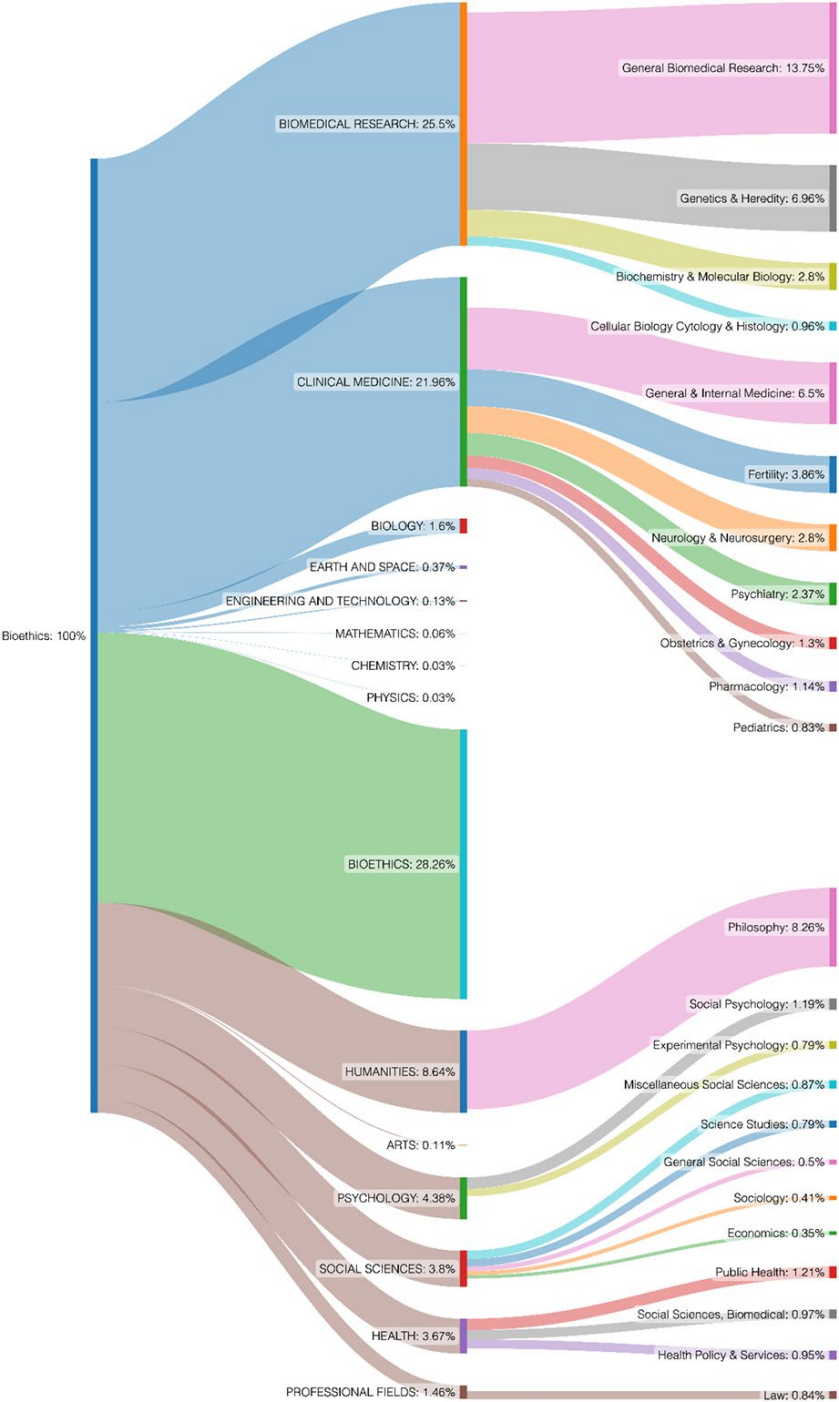
presents the citation flow (10,946 references) from all 1,360 documents in the ENHANCEMENT subcorpus to documents in journals representing two main branches of science (NSE disciplines are represented by the blue color on the left-hand side of the figure, whereas SSH disciplines are represented by brown), scientific disciplines (e.g., BIOMEDICAL RESEARCH) and subdisciplines (e.g., General Biomedical Research). For the sake of clarity, the right-hand side of this figure presents only the most popular subdisciplines

Figure 1 presents the citation flow outgoing from the ENHANCEMENT subcorpus (10,946 citations from 1,360 documents) to journals indexed by the Web of Science.^16^ Notably, almost half (5,438, 49.6%) of the references point to journals classified as Natural Science and Engineering (NSE), which can be further divided into the disciplines of BIOMEDICAL RESEARCH (BR) (25.5%) and CLINICAL MEDICINE (CM) (21.96%), with a negligible role of other disciplines (BIOLOGY 1.6%, EARTH AND SPACE 0.37%, ENGINEERING AND TECHNOLOGY 0.13%, and almost no citations to MATHEMATICS, CHEMISTRY, PHYSICS). More than a quarter of the citations go to BIOETHICS (28.26%) and the rest to Social Science and Humanities (SSH) (22%), including disciplines of the HUMANITIES (8.6%; predominantly Philosophy journals: 8.26%), and less to PSYCHOLOGY (4.38%), SOCIAL SCIENCES (3.8%), HEALTH (3.67%), and PROFESSIONAL FIELDS (1.46%, including Law journals: 0.84%).

The results for the citation flow outgoing from the whole corpus of seven main bioethics journals (178,845 citations from 19,488 documents)^17^ are very similar (BIOETHICS 26.8%; SSH: 23.5%, including Philosophy 5.9%), with one important exception: Although NSE journals also receive almost half (49.7%) of the citations from seven main bioethics journals, the discipline BIOMEDICAL RESEARCH receives only 7.63%, whereas CLINICAL MEDICINE receives 41.31%, with the negligible role of other NSE disciplines.

### The Visibility of Bioscience in the ENHANCEMENT Subcorpus

The fact that a document from the ENH subcorpus cites a paper published in a journal classified as NSE does not imply a reference to novel scientific findings. This is so because a significant number of these citations go to NSE documents whose main aim is to present purely normative, conceptual, or regulatory claims. This is the case of the three documents from the NSE journals most cited by the ENH subcorpus (Baltimore et al., 2015; Lanphier et al., 2015; Baylis, 2013). They were published, using the journals’ own terminology, as, respectively, ‘Perspectives’, ‘Commentary’, and ‘Comment’. Table 2 presents the 10 documents from the NSE journals most frequently cited by the ENH subcorpus. Only four of them report new empirical research (E), while six present purely normative claims (N).

**Table 2:** The 10 most-cited documents by the ENH cluster published in Natural Science and Engineering (NSE) journals (editorial materials = EM; article = A; review = R; normative = N; empirical = E). Total citations in WoS were counted on 3 November 2024

	Title	The first author	Journal	Year	Citations from ENH in WoS	Total citations in WoS	Doc. Type in WoS	Content type
1	A prudent path forward for genomic engineering and germline gene modification	Baltimore, David	*Science*	2015	22	441	EM	N
2	Don’t edit the human germ line	Lanphier, Edward	*Nature*	2015	20	343	EM	N
3–4	The ethics of creating children with three genetic parents	Baylis, Françoise	*Reproductive BioMedicine Online*	2013	18	99	EM	N
3 − 4	CRISPR/Cas9-mediated gene editing in human tripronuclear zygotes	Liang, Puping	*Protein & Cell*	2015	18	759	A	E
5–7	Deaf lesbians, “designer disability,” and the future of medicine	Savulescu, Julian	*BMJ*	2002	16	108	EM	N
5 − 7	Correction of a pathogenic gene mutation in human embryos	Ma, Hong	*Nature*	2017	16	652	A	E
5 − 7	New breeds of humans: The moral obligation to enhance	Savulescu, Julian	*Reproductive BioMedicine Online*	2005	16	83	EM	N
8	Serotonin selectively influences moral judgment and behavior through effects on harm aversion	Crockett, Molly J.	*PNAS*	2010	13	348	A	E
9	Pronuclear transfer in human embryos to prevent transmission of mitochondrial DNA disease	Craven, Lyndsey	*Nature*	2010	12	340	A	E
10	Neurocognitive enhancement: What can we do and what should we do?	Farah, Martha J.	*Nature Reviews Neuroscience*	2004	11	376	R	N

The most-cited document from the NSE journals by Baltimore et al. (2015) is an influential editorial material suggesting, on the one hand, discouragement of “any attempts at germline genome modification for clinical application in humans”, albeit, on the other hand, “enabl[ing] pathways to responsible uses of this technology”, i.e., CRISPR/Cas9. The commentary was published in *Science* in April 2015, around the time when the first embryo experiments in China were conducted (Liang et al., 2015). The editorial aims at steps necessary to ensure “that the application of genome engineering technology is performed safely and ethically”, and its optimistic tone regarding the applications of CRISPR/Cas9 contrasts with the previous consensus against any germline modification and even with the commentary by Lanphier et al. (2015) published in *Nature* in March 2015, which is the second-most-cited document from the NSE journals and calls for “a voluntary moratorium… to discourage human germline modification”. Other editorial or normative papers from the group of the ones most cited concern the evaluation of mitochondrial replacement techniques (Baylis, 2013), parental wishes to select for disability (Savulescu, 2002b), a call to “enhance human beings” because of moral reasons (Savulescu, 2004), and dealing with ethical issues regarding neurocognitive enhancement (Farah et al., 2004).

Not surprisingly, many of the most-cited empirical articles from NSE journals concern germline genome editing in human embryos, which is an ethically controversial procedure. For example, the paper by Liang et al. (2015), which shares the 3rd and 4th place of the most-cited documents published in NSE journals, presents results from the first experiments on (non-viable) human embryos, and the paper by Ma et al. (2017), which shares the 5th to 7th place, describes introducing genetic changes in human embryos and is considered to mark a shift to the clinical application of germline genome editing. Some of the most-cited NSE papers concern mitochondrial replacement techniques (Craven et al., 2010 at the 9th position; Tachibana et al., 2009 at the 11th; and Yamada et al., 2016, at 17th ). The ‘classical’ papers on cloning (Wilmut et al., 1997) or CRISPR/Cas9 (Jinek et al., 2012) are classified at the 15th and 21st positions, respectively. Some empirical papers from this group use biomedical interventions to measure behavioral changes, for example the paper on the influence of serotonin on moral judgment and behavior by Crockett et al. (2010), at position 8, and the paper by Dreu et al. (2011), at position 13, who reported that the type of altruism promoted by oxytocin seems to be ‘parochial’ and ‘ethnocentric’. Other empirical papers in this group reported that oxytocin improved memories in mice (Tang et al., 1999, p. 19), led to an increase in levels of trust and cooperation between people (Kosfeld et al., 2005, p. 20), or reported a correlation between levels of monoamine oxidase A (MAOA) activity and various measures of antisocial behavior (Caspi et al., 2002, p. 22). Notably, the most-cited NSE papers are cited relatively rarely in our ENH subcorpus (the first in the ranking is cited only 22 times, while the paper in the 10th position is cited 11 times, and the paper in the 20th position is cited 8 times), in comparison to the most frequently cited papers (e.g., Savulescu’s paper on procreative beneficence (2002a) was cited 80 times from the ENH subcorpus).^18^

### Types of Engagement with Scientific Journals

Then we tried to analyze the type of content from scientific journals that influences bioethics. Since the analyzed set consists of 10,946 citations (including 5,438 citations to NSE journals), it would be impractical to read all the cited documents to check what type of scientific content they provide. However, we decided to check two sets of references outgoing from the ENH subcorpus to journals classified as NSE (by analyzing their titles, abstracts, and, when necessary, the full content): The first one consisted of the 100 most-cited documents from NSE journals; the second one was a random sample of 100 cited documents from NSE journals.^19^ The reason for this choice was that we wanted to gain insight into content of not only typical papers cited from NSE journals, but also into that of the most influential papers. However, because of the high dispersion of citations outgoing from the ENHANCEMENT corpus, the papers at the bottom of our list of the 100 most-cited documents from NSE journals received only three citations.

**Fig. 2:**
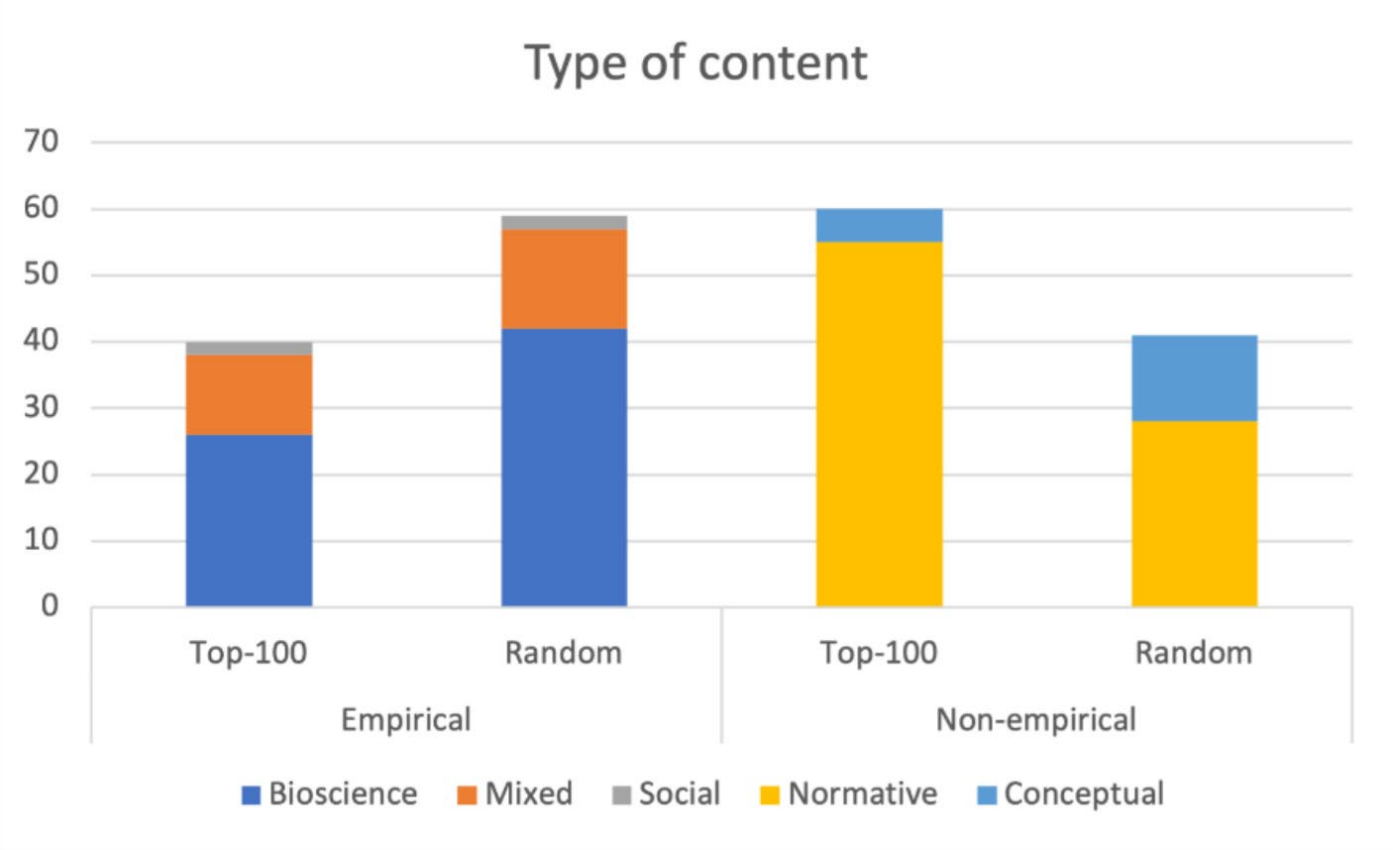
presents the types of content in the 100 most-cited documents and in the random sample of 100 papers

We classified all the texts in these samples either as Empirical or Non-empirical. Further, we distinguished three types of Empirical content, i.e., Bioscience, Mixed, and Social,^20^ as well as two types of Non-empirical content: Normative and Conceptual.^21^ Sixty out of the 100 most-cited documents had Non-empirical content, and only 40 were empirical (including 26 biomedical, 12 mixed, and 2 social). However, in the random sample, the proportions were reversed: 59 documents had empirical content (including 42 biomedical, 15 mixed, and 2 social), while 41 were Non-empirical. The results are presented in Fig. 2.

**Fig. 3:**
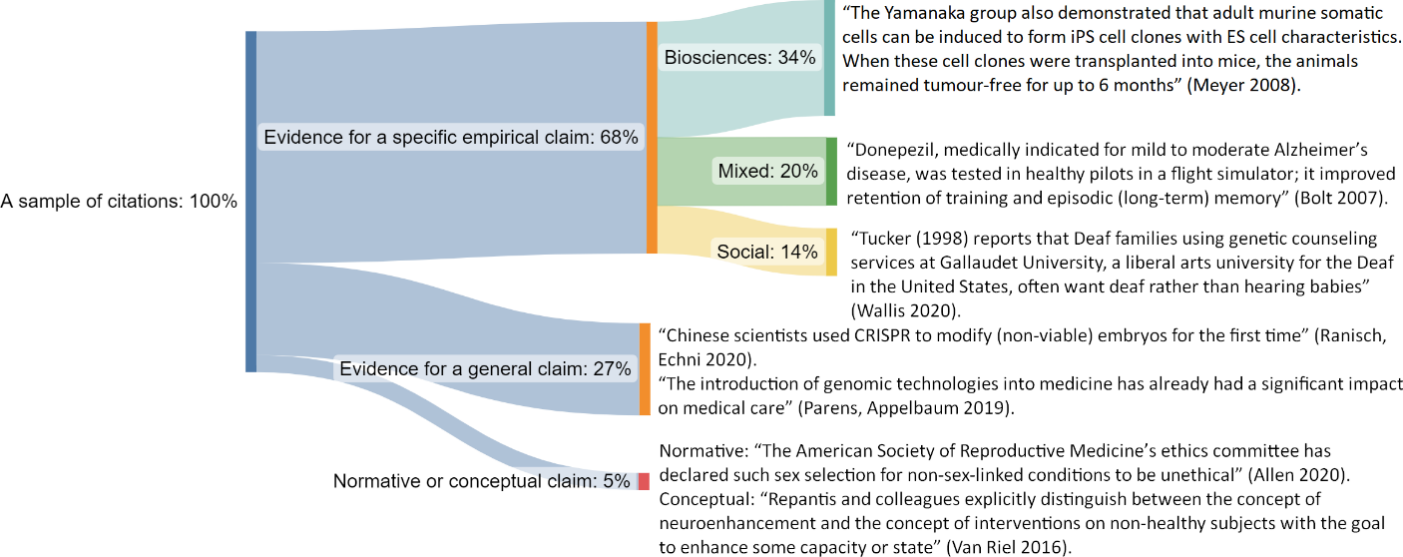
presents the main types of engagement of bioethical literature with empirical texts published in the NSE journals on the basis of the analyses of the citation context in the sample of 59 ENH papers (all citations of empirical papers in a random sample of 100 outgoing references). We provide examples of quotations from: Meyer, 2008; Bolt, 2007; Wallis, 2020; Ranisch & Echni, 2020; Parens & Appelbaum, 2019; Allen, 2020; Van Riel, 2016

To analyze the type of interaction between bioethics and empirical literature in the NSE, we carefully read citation contexts (i.e., sentences just before and after the citation) in all 59 ENH papers that refer to the empirical articles from the random sample. The citation contexts were analyzed by one of the authors (TŻ), and, in eight problematic cases, the verdict was consulted with another coauthor (VD). Taking into account the specificity of bioethics, we distinguished three types of engagement with the empirical literature: evidence for a specific empirical claim, evidence for a general claim, and reference to some normative or conceptual claim.^22^ The first type cites an empirical paper with the aim of discussing, or at least mentioning, a specific empirical study or discovery reported in the cited paper. In contrast, the general approach does not use cited articles to refer to any specific evidence, but rather to state that a study has been undertaken or to express a very general claim or truism that has not been established specifically within the research reported by the cited paper. In the random sample (59 papers), evidence for a specific empirical claim was the most common reason for citation (40 papers, which amounts to 68%, with two others accounting for 27% and 5%, respectively). Furthermore, in the first category, we distinguished between three types of specific empirical claims: biosciences (20 papers, 34%), mixed (12 papers, 20%), and social (8 papers, 13%).^23^ Figure 3 presents types of references with typical examples of citation contexts.

We also checked the average citation rate in 5,438 ENH citations that referred to NSE papers, which was relatively lower (1.27) than in the case of citations that referred to BIOETHICS itself (2.23). As many as 4,195 different NSE articles were cited from the ENH subcorpus,^24^ and 86% of the NSE articles cited by our ENH subcorpus were cited only once (in the case of BIOETHICS itself, 1,388 different BIOETHICS articles were cited, and only 63% of the BIOETHICS articles cited by our ENH subcorpus were cited only once).

Moreover, there is no document published in NSE journals that would be cited by papers published in all seven core bioethics journals.^25^ For example, famous papers reporting the first uses of CRISPR/Cas9 germline modifications on human embryos (Liang et al., 2015; Ma et al., 2017) have not been cited in *JMP*, *TMB*, or *MHCP*. The only three documents cited by papers from the ENH subcorpus and published in all seven journals belong either to BIOETHICS itself: ‘Procreative beneficence: Why we should select the best children’ (Savulescu, 2002a) and ‘In defense of posthuman dignity’ (Bostrom, 2005) or to Philosophy: ‘Wrongful life and the counterfactual element in harming’ (Feinberg, 1986).

Finally, although one could expect the ENH cluster to be especially likely to cite the latest research in the disciplines of BIOMEDICAL RESEARCH and CLINICAL MEDICINE, the mean age of the papers cited by the ENH cluster is very similar to the average for all corpus: The mean age of the papers (i.e., the difference between the publication dates of the citing article and the cited article) from BIOMEDICAL RESEARCH and CLINICAL MEDICINE (the two most relevant NSE disciplines) cited by the ENH subcorpus is 8.38 years, whereas in the case of our whole corpus it is 8.55 years. Moreover, the mean ages of the other papers cited by both the ENH subcorpus and our whole corpus are slightly older (9.10 years and 9.36 years, respectively).

## Discussion

Our analyses offer a nuanced view of the complex engagement of bioethical discourse on human enhancement with NSE papers. Although citations are commonly treated as indicators of some form of communication between researchers, the raw citation flow, as presented in Fig. 1, may be misleading. First, apart from the limitations of any categorization that maps journals to disciplines, the raw citation count overestimates the *scale* of engagement of the ENH subcorpus with discoveries in biosciences. The main reason is that a significant portion of the citations to NSE journals (41% if we accept results from our random sample, although this may be significantly higher in the most-cited papers) do not refer to any empirical studies, but rather to texts with normative content, predominantly editorial materials, very often written by bioethicists themselves. Although references to such content are not evidence for engagement with empirical studies, they nevertheless demonstrate that the editors of NSE journals are open to bioethical issues and that bioethicists themselves do not shy away from engaging with those issues and reaching out to scientists in places that are likely to be read by them.

Second, given that there are different reasons for citations, the raw citation counts may give a distorted vision of the *type* of engagement of the ENH subcorpus with discoveries in biosciences. In particular, our study shows that often, even if a citation refers to an empirical paper, it does not refer to any specific scientific discovery or study, but refers either to the justification of some very general claim (not necessarily stated in the cited paper) or to a normative claim or conceptual distinction. In the analyzed random sample, this was the case in one-third of citations of empirical papers (19 out of 59). In particular, cases of references to NSE journals that only justify some general claims (16 out of 59) cannot be treated as serious engagement with the empirical literature. In many such cases, BIOETHICS papers refer to empirical studies only to acknowledge the existence of controversial experiment: “The team of Shoukhrat Mitalipov from Oregon Health and Science University used genome editing technology (See also Jinek et al., 2013) to perform gene editing on human embryos on July 26, 2017, the first in the United States” (Šlesingerová, 2019). Moreover, some of the citations of this type are ‘unsubstantiated’ because the information contained in the citation context contradicts, is unrelated to, or is simply different from the reference. This observation aligns with the results of these bibliometric studies, which highlighted a generally high proportion of ‘unsubstantiated’ citations in the main science journals (Smith, & Cumberledge, 2020), and there is no reason to assume that bioethics as a discipline is an exception in this regard.

Third, regarding the specificity of bioethics, even if a citation refers to an empirical paper and a specific bioscientific discovery (as in the case of 20 papers in our random sample of 100), it does not necessarily indicate the intellectual *influence* of the cited research on the new study. Conceptualizing such influence is particularly problematic in bioethics, which often focuses not on the technical details of the discovery itself, but on conceptual, normative, or regulatory issues related to scientific and medical practice. An additional difficulty arises from the interdisciplinarity of such influence.

To provide a particularly vivid example of what we mean by such interdisciplinary intellectual influence, consider Lee (2022), a philosophy paper that ingeniously uses recent discoveries about human embryo development to reach a metaphysical conclusion about an object’s identity over time (although we do not discuss here how convincing this argument in fact is). The main claim refers to experimental studies in which blastomeres of a 16-cell mouse embryo were separated and then reaggregated at random, switching their positions. The fact that the reconstituted embryos developed into a fertile mouse (Suwińska et al., 2008; see a similar experiment on human embryos: De Paepe et al., 2013) was crucial for the main conclusion that a zygote that naturally developed into a singleton could instead have developed into a numerically distinct singleton. Lee argues that this has an important philosophical implication: a human infant is numerically distinct from the zygote from which it originated.

Analogically, one could expect the most important recent discoveries in bioscience to influence the way a bioethicist conceptualizes some theoretical and normative issues, for example concerning regulatory framework of research with human participants. To check whether this indeed happened, we analyzed all references to the most-cited empirical paper, which is about the use of CRISPR/Cas9 to cleave and then repair the β-globin gene (HBB) gene in nonviable human embryos (Liang et al., 2015). We found that a significant number of citations for this paper just neutrally refer to the fact that this study has been conducted while discussing rather abstract topics, such as why “many instances of genome editing will be moral imperatives” (Gyngell et al., 2019; see also Evitt et al. (2015), who discuss a model regulatory framework for CRISPR/Cas9 germline editing therapies). Other documents noted some ethical controversies around this study (“To say the experiment prompted controversy is an understatement” (Scott, 2015; see also Ranisch & Ehni, 2020, who noted that the experiment “not only raised public concerns around the globe but also alarmed parts of the scientific community”). Most interestingly, this paper was also treated as providing empirical evidence for the normative conclusion: “it almost went unnoticed that their [Liang et al.’s (2015)] research confirmed exactly the point that their opponents were making: that genome editing in embryos should at present not be used for clinical applications due to inefficiency, mosaicism, off-target ‘edits’, and so on” (Mertes & Pennings, 2015; see also Evitt et al., 2015), which repeats a conclusion explicitly expressed in the original publication: “Clinical applications of the CRISPR/Cas9 system may be premature at this stage” (Liang et al., 2015, p. 364).

Furthermore, the lack of a list of scientific texts that would be cited frequently and/or by all (or at least most) BIOETHICS journals, as well as the fact that the great majority (86%) of the NES articles cited by our ENH subcorpus were cited only once, is difficult to interpret. On the one hand, it can be evidence that bioethicists do not perform perfunctory citations focused on a few high-prestige publications, but rather always cite the study most relevant to their claim, even if little is known. On the other hand, from our study of the random sample one can extrapolate that only approximately 20% of all citations from the ENH subcorpus refer to evidence for a specific empirical claim in the bioscience sense (and additionally 12% in the mixed sense and 8% in social sense), which may be interpreted as a general lack of interest in real-life discoveries in biosciences.

Finally, given that bioethics is a practice-oriented discipline that involves bringing moral reason to bear upon pressing practical issues (McMillan, 2018), one could expect that it is much less self-referential than the more mature and well-established disciplines within the social sciences, such as sociology or economics. One may justify this expectation by the very practical and regulatory aims of bioethics, which aspires to present guidance regarding discoveries in biotechnology, biomedical research, and clinical practice (Evans, 2012). Data presented by Wallace et al. (2012), who used the same NSF journal classification as we did, show that the degree of self-referentiality, measured by the raw citation flow to other journals in the same discipline, is indeed greater in most parts of the humanities and social sciences in the last 20 years (e.g., sociology – approximately 40%; history – approximately 55%; economics – approximately 70%) than in the case of BIOETHICS in the ENH cluster (28%). However, if one treats references to normative articles in NSE journals as evidence for the self-referentiality of this part of bioethics, this number will be much greater, and self-referentiality of bioethics may not be much lower than that of more ‘mature’ disciplines. One may reply by arguing that our study concerns scholarly theory-oriented bioethics. For example, in a well-known trichotomy proposed by Battin, bioethics may be understood either as “theoretical reflection, grounded in philosophical inquiry” or as clinical consultation or as policy development (Battin, 2013, p. 2).

In contrast to Battin’s definition and Harris’s (2011) expectation that the bioethical discourse on human enhancement combines “cutting-edge science with mainstream philosophy”, this part of bioethics does not appear to be firmly “grounded in philosophical inquiry”. Although over 8% of citations from the ENH subcorpus refer to Philosophy journals, the ENH cluster generally shows little interest in central philosophical questions. Many of the cited papers from Philosophy journals address issues internal to the bioethics debate on enhancement rather than typical philosophical problems. This trend is also evident in the entire corpus of seven main bioethics journals, where only 6% of outgoing citations target Philosophy journals.^26^ Iltis (2023) recently highlighted a general issue with philosophical approaches in bioethics, noting that it “can undermine the practical goals of the bioethics project.” However, this explanation does not seem applicable to the bioethical discourse on human enhancement, which has garnered significant interest not in real-life dilemmas, but in futuristic techniques such as genetic modification of complex traits, human–machine–neural interfaces, and other potential advancements in pharmacology and nanotechnology that could enhance human moral or cognitive capabilities. As an example, none of the most prominent papers in the analyzed topics of *Enhancement* and *Ecology* address existing technologies; they focus instead on speculative possibilities like moral enhancement or the de-extinction of certain animal species. The topics of the most-cited papers in our corpus suggest that this segment of bioethics is primarily concerned with concepts developed within the discourse on human enhancement itself, such as procreative beneficence (Savulescu, 2002a), posthuman dignity (Bostrom, 2005), and moral enhancement (Harris, 2011). Additionally, it addresses specific issues related to (as yet non-existent) enhancement technologies, including equality (Sparrow, 2014) and the expected obsolescence of enhancement technologies (Sparrow, 2019).

## Conclusion

It is sometimes claimed that the institution of public bioethical debate was established to regulate the actions of scientists and medical practitioners, as well as to create a straightforward ethical framework for research that could be incorporated into public law (Evans, 2020). The primary aim of the Belmont Report was to establish principles – autonomy, beneficence, non-maleficence, and justice – that are generally accepted for many cultural traditions and should underlie the conduct of biomedical and behavioral research involving human subjects (National Commission, 1978). Our study demonstrates how current trends in some parts of bioethics have diverged significantly from the discipline’s origins. Namely, while approximately half of the outgoing citations are to papers published in journals of medicine and biomedical sciences, almost half of these citations cite non-empirical papers, and there are very few highly cited empirical papers. Even within empirical citations, there are many citations that are used for supporting general or normative claims rather than specific claims. Our interpretation of these results is that rather than closely monitoring recent discoveries in biomedicine that could be even more profound than those that lay the ground for the beginnings of bioethics, the segment of bioethics that deals with human enhancement focuses on eugenic fantasies of improving traits such as intelligence and empathy in humans. This focus suggests that a significant portion of the bioethical discourse on human enhancement may be influenced by “science-fictional habits of mind” (Clayton, 2013).

## Footnotes

1 The method was recently used to analyze diachronic changes in some disciplines, including general philosophy (Weatherson, 2022), philosophy of science (Malaterre et al., 2021), or to track patterns of value change (de Wildt et al., 2022).

2 Citation flow was recently used by Weingart (2015) to analyze the connection between history of science and philosophy of science; by Bonino et al. (2022) to analyze the way philosophers used formal research methods such as game theory and network theory; by Pradeu et al. (2024) and Khelfaoui et al., (2021) to assess the visibility of philosophers in scientific journals; and by Yan and Liao (2023) to measure cross-disciplinarity in the empirically informed philosophy of mind. It is worth noting that our aim is somewhat the reverse of Pradeu et al. (2024): We want to measure the ‘permeation’ of (bio)sciences in bioethics journals, rather than the ‘permeation’ of bioethicists in (bio)science journals.

3 *Bioethics*; *Journal of Medical Ethics* (*JME*); *American Journal of Bioethics* (*AJOB*); *Medicine*,* Health Care and Philosophy* (*MHCP*); *Hastings Center Report* (*HCR*); *Journal of Medicine and Philosophy* (*JMP*); and *Theoretical Medicine and Bioethics* (*TMB*). The details of the procedure are described here: Bystranowski et al., (2022).

4 We *italicize* the names of the topics in this technical sense.

5 This website enables checking every document in the corpus: https://bioethics.incet.uj.edu.pl/documents.

6 See the list of the top-10 papers most characteristic for this and other topics: https://bioethics.incet.uj.edu.pl/top10-documents-per-topic.

7 For all overall changes in topic prominence, see the online supplement: https://osf.io/9pu5h.

8 For all intertopic correlations, see the online supplement: https://osf.io/eqjsm.

9 We have omitted the topic most correlated with *Enhancement*, that is, *Moral philosophy*, because it represents one of ‘framing topics’, that is, topics that represent distinct ways in which content topics are ‘framed’ in a given document (see: Bystranowski et al.,^2022^).

10 More technically: for which the sum of probabilities of assignment to each of our five topics was at least 0.15. Here is the list of all 1,360 papers that fall under the ENH subcorpus: https://osf.io/2qe6t?view_only=418d5e72caf341e28800ae71cdf62e75.

11 We capitalize name of ENHANCEMENT (ENH) cluster which refers to the set of five *topics*, which we italicize.

12 https://osf.io/jgd8t?view_only=418d5e72caf341e28800ae71cdf62e75.

13 However, we implemented some minor modifications: We moved philosophy-of-science journals (e.g., *Philosophy of Science*, *Synthese*, etc.) and some other clearly philosophical journals (e.g., *Ethics*, *Philosophy & Public Affairs*, etc.) to the category Philosophy. Here is the list of all changes: https://osf.io/znyb7?view_only=418d5e72caf341e28800ae71cdf62e75.

14 We capitalize the names of disciplines when referring to the NSF classification used in our study (e.g. BIOMEDICAL RESEARCH or HUMANITIES), including our addition of the discipline BIOETHICS, and we capitalize the first letters of the names of subdisciplines (e.g., General Biomedical Research or Philosophy).

15 *BMC Medical Ethics; Cambridge Quarterly of Healthcare Ethics; Developing World Bioethics; Health Care Analysis; HEC Forum; Journal of Bioethics Inquiry; Journal of Clinical Ethics; Journal of Empirical Research on Human Research Ethics; Journal of Law and the Biosciences; Journal of Law*,* Medicine & Ethics; Kennedy Institute of Ethics Journal; Neuroethics; Nursing Ethics; Public Health Ethics*. The main reason for this was to avoid the situation when citations between the ENH cluster and clearly bioethics journals (e.g., from *Bioethics* to *Cambridge Quarterly of Healthcare Ethics*) are counted as citations between the ENH and HEALTH/Health Policy & Services to which most of these bioethics journals belong in the NSF classification.

16 The citation flow outgoing from the ENHANCEMENT subcorpus: https://osf.io/qprg3?view_only=418d5e72caf341e28800ae71cdf62e75.

17 The citation flow outgoing from the whole bioethics corpus: https://osf.io/8pz5h?view_only=418d5e72caf341e28800ae71cdf62e75.

18 For comparison, see the list of the most-cited papers in BIOETHICS and Philosophy (Tables 1 and 2 in Appendix 1: https://osf.io/jgd8t/?view_only=418d5e72caf341e28800ae71cdf62e75).

19 Here is the result of our classification of the list of a random sample of 100 documents from NSE journals cited by the papers from the ENH subcorpus: https://osf.io/kfrv9?view_only=418d5e72caf341e28800ae71cdf62e75.

20 Here are paradigmatic examples: Ma et al. (2017), who corrected the heterozygous MYBPC3 mutation in human preimplantation embryos with the CRISPR–Cas9 tool, is a typical empirical bioscience paper; Dreu et al. (2011), who measured the behavioral effects (ethnocentrism) of a peptide hormone oxytocin, was classified as mixed paper; Baruch et al. (2008), who explored the attitudes and opinions of clinic directors toward preimplantation genetic diagnosis (PGD), was classified as social-science paper.

21 Paradigmatic examples are, respectively, Lanphier et al. (2015) and Earp et al. (2014).

22 A recent review enumerated 13 reasons for citations (Tahamtan & Bornmann, 2018, p. 208), but most of these are not relevant in the bioethical context. See also Tahamtan and Bornmann (2019) for a review of research on citation flows.

23 The citation context in 59 analyzed papers can be found at the top of this document: https://osf.io/n9tsz?view_only=418d5e72caf341e28800ae71cdf62e75. Additionally, we checked the group of the randomly selected 40 ENH papers that refer to the 40 most-cited empirical documents from NSE. In this group, the shares were, respectively, specific claim: 37,5%; general claim: 52,5%; normative or conceptual claim: 10% (see the bottom of the document linked above).

24 In the case of 82 citations, the titles of cited papers were missing in our data. In the whole corpus, the average citation rate in 88,855 citations that refer to NSE journals was higher (2.06), since 43,208 different articles were cited, and 75% of cited papers were cited only once. However, the comparison between the whole corpus and the ENH subcorpus is problematic because in the larger corpus one can easily expect the average citation rate to be higher.

25 So there is no ‘knowledge core’ as defined by Bonino et al. (2022), that is, there is no reference to a science paper that would appear in the reference list of all journals included in the ENH subcorpus.

26 https://osf.io/8pz5h?view_only=418d5e72caf341e28800ae71cdf62e75.
