# Correction: Discussions on Human Enhancement Meet Science: A Quantitative Analysis

**DOI:** 10.1007/s11948-025-00537-0

**Published:** 2025-03-18

**Authors:** Tomasz Żuradzki, Piotr Bystranowski, Vilius Dranseika

**Affiliations:** 1 Jagiellonian University, Institute of Philosophy & Interdisciplinary Centre for Ethics, ul. Grodzka 52, Kraków, 31-044 Poland; 2 Jagiellonian University, Interdisciplinary Centre for Ethics, ul. Grodzka 52, Kraków, 31-044 Poland; 3 Max Planck Institute for Research on Collective Goods, Bonn, Germany

**Science and Engineering Ethics (2025) 31:6**

10.1007/s11948-025-00531-6

In this article the statement in the Funding information section was incorrectly given as

‘European Research Council (ERC) under the H2020 European Research Council Research and Innovation Program, Grant/Award Number: 80549’

and should have read

‘European Research Council (ERC) under the H2020 European Research Council Research and Innovation Program, Grant/Award Number: 805498. The purchase of access to the Web of Science API has been supported by a grant from the Faculty of Philosophy under the Strategic Programme Excellence Initiative at Jagiellonian University. This article is being published in open access with funding support from the Faculty of Philosophy under the Strategic Programme Excellence Initiative at Jagiellonian University’.

The original article has been corrected.

